# Application of single and cooperative different delivery systems for the treatment of intervertebral disc degeneration

**DOI:** 10.3389/fbioe.2022.1058251

**Published:** 2022-11-14

**Authors:** Zongtai Liu, Changfeng Fu

**Affiliations:** ^1^ Department of Orthopedics, Affiliated Hospital of Beihua University, Jilin, China; ^2^ Department of Spine Surgery, First Hospital of Jilin University, Changchun, China

**Keywords:** delivery system, intervertebral disc degeneration, biomaterial, nanometer materials, low back pain

## Abstract

Intervertebral disc (IVD) degeneration (IDD) is the most universal pathogenesis of low back pain (LBP), a prevalent and costly medical problem across the world. Persistent low back pain can seriously affect a patient’s quality of life and even lead to disability. Furthermore, the corresponding medical expenses create a serious economic burden to both individuals and society. Intervertebral disc degeneration is commonly thought to be related to age, injury, obesity, genetic susceptibility, and other risk factors. Nonetheless, its specific pathological process has not been completely elucidated; the current mainstream view considers that this condition arises from the interaction of multiple mechanisms. With the development of medical concepts and technology, clinicians and scientists tend to intervene in the early or middle stages of intervertebral disc degeneration to avoid further aggravation. However, with the aid of modern delivery systems, it is now possible to intervene in the process of intervertebral disc at the cellular and molecular levels. This review aims to provide an overview of the main mechanisms associated with intervertebral disc degeneration and the delivery systems that can help us to improve the efficacy of intervertebral disc degeneration treatment.

## Introduction

Low back pain (LBP) is a widespread health problem characterized by a long duration and high disability rate that is a cause of widespread concern (Katz, 2006). LBP seriously affects the quality of life and is one of the top three causes of disability in developed countries (Murray et al., 2012; Hartvigsen et al., 2018). According to past statistics, it is estimated that LBP affects almost 700 million people worldwide, although this number is increasing (GBD 2017 Disease and Injury Incidence and Prevalence Collaborators, 2017; Jin et al., 2020). LBP also imposes a significant burden on society and the economy. LBP is responsible for more than 30% of work absences and its directly related costs are as high as $90 billion annually in the United States alone (Dieleman et al., 2016; Hartvigsen et al., 2018).

The etiology of LBP is complex and has yet to be fully elucidated. The current consensus is that LBP is caused by multiple factors, including biological, psychological and social factors (Vlaeyen et al., 2018). Despite many cognitive limitations, Intervertebral disc (IVD) degeneration (IDD) is definitely an important cause of LBP (Cheung et al., 2009; Mohd Isa et al., 2022). Interestingly, not all patients with IDD develop LBP. A previous study estimated that between 26% and 42% of IDD patients experience significant LBP (Peng, 2013; Mohd Isa et al., 2022). There are two main possible reasons for such pain, one is the sagittal imbalance of the spine due to disc height reduction which may lead to soreness from exertion of the surrounding muscles; the other reason is the inflammation caused by damage to the disc and its adjacent tissues.

The three-joint structure between adjacent vertebral bodies plays an important role in motion, weight-bearing, maintaining flexibility and protecting vulnerable tissues. IVDs are a crucial fibrocartilage component of this structure and regulate the amounts of bound water to ensure that the intrinsic intradiscal pressure is within the appropriate range (Urban and McMullin, 1988; Vergroesen et al., 2014). The intradiscal pressure is approximately 0.1 MPa–2.3 MPa according to its loading (Sato et al., 1999; Wilke et al., 1999). The ability to retain water under stress decreases in degenerating IVDs; this is reflected by the reduction of disc high and intradiscal pressure (Iatridis et al., 2013; Lee et al., 2013; Vergroesen et al., 2014). At the same time, the reduced intradiscal pressure increases the shear stress concentrations in IVDs, thus leading to subsequent complications such as herniation (Adams et al., 1996; Andersson et al., 2006). The main anatomical structure of an IVD consists of three parts, the nucleus pulposus (NP), the annulus fibrosus (AF) and the cartilaginous endplates (CEP). The healthy NP is a highly hydrated tissue that is rich in proteoglycans and generates an intradiscal pressure and evenly distributes pressure on the adjacent endplates (Iatridis et al., 2013; Vergroesen et al., 2014). The AF is composed of collagen fibers, which together with lamellae and proteoglycans, form a multilayered concentric structure (Nerurkar et al., 2010). The AF is tensioned by intradiscal pressure and also plays a role in protecting and constraining the NP inside it (Iatridis et al., 2013). It is worth noting that the majority of the AF is avascular and nerve-free, thus making its renewal and healing highly dependent on sparsely distributed cell populations. At the same time, fragile blood supply and nutritional support mean that once damaged, the AF experiences difficulty in repairing itself (Holm et al., 1981). In a healthy state, the endplate is composed of homogeneous hyaline cartilage of uniform thickness (Rutges et al., 2013). Almost all nutrients are transported to the IVD via the CEPs (Malandrino et al., 2014; Bowles and Setton, 2017). Cartilage damage is an important characteristic of IDD, and is a similar condition to osteoarthritis (Wang et al., 2012; Zhu et al., 2022b; Francisco et al., 2022). Mineralized endplates may hinder nutrient transport to the IVD, and insufficient nutrient supply has been considered as one of the causes of IDD (Roberts et al., 1996; Benneker et al., 2014; Huang et al., 2014; Malandrino et al., 2014). Collectively, the IVD components can affect each other and any single site of injury or degeneration can lead to overall IDD.

In clinical work, magnetic resonance imaging (MRI) of the spine is the most commonly used method to detect IDD. There are some IDD classification methods based on T2-weighted MRI. Pfirrmann divided IDD into five grades (grade I to grade V) according to structural heterogeneity, the degree of differentiation between the NP and AF, disc height and signal density (Pfirrmann et al., 2001). In 2007, Griffith made an adjustment based on the previous classification by classifying IDD into eight grades (grade I to VIII). Unfortunately, T2-weighted MRI has irreparable limitations in detecting the early stage of IDD. Bruno et al. (Zobel et al., 2012) used T1ρ to identify early IDD and found that this system achieved good discrimination performance. In addition, more advanced equipment, along with the development of artificial intelligence (AI) technology, provide new directions for the detection of IDD (Sher et al., 2019; Gao et al., 2021). At present, there are three main approaches to treating LBP caused by IDD, including pharmacological therapies, physical and psychological therapies, and surgical interventions (Foster et al., 2018). Nevertheless, all of these approaches are remedial for patients with IDD who already have obvious symptoms. Blocking disease progression at an early stage can further reduce symptoms and complications and improve quality of life. Benefiting from the rapid development of modern biotechnology, an increasing number of biomarkers have been detected, thus making it possible for us to intervene in the development of IDD. However, an inevitable problem is how to deliver the required substances to the appropriate location to achieve effective intervention. Therefore, in the present study, we review the mainstream understanding of IDD-related pathological mechanisms and summarize the delivery systems that could effectively intervene in these mechanisms. Furthermore, we analyzed the classification, application and clinical application prospects of these systems.

## The pathophysiology of intervertebral disc degeneration

**FIGURE 1:**
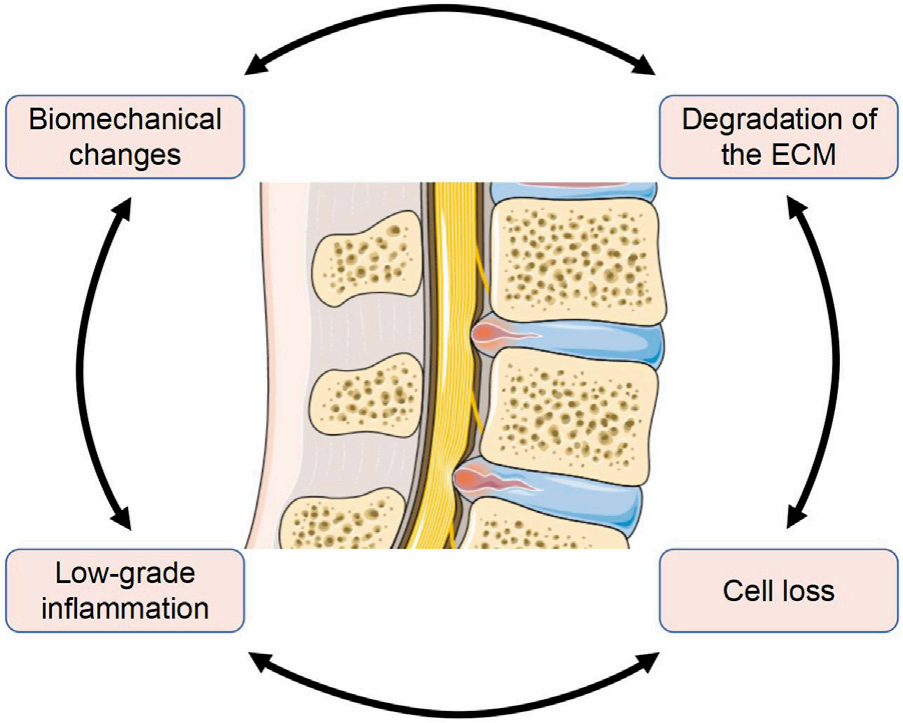
Several major pathological mechanisms in IDD are interconnected (The Figure was partly generated using Servier Medical Art, provided by Servier, licensed under a Creative Commons Attribution 3.0 unported license).

The pathophysiology of IDD is intricate and has yet to be explained fully. Several pathological changes of IVD are thought to be related to IDD. These pathological mechanisms involve almost all levels of the IVD, from the microscopic level such as gene and cytokine expression to the macroscopic level such as structural loading and composition changes. These changes are interconnected and create a vicious cycle (Figure 1). Enhancing our understanding of the pathological processes involved will provide new opportunities and challenges for the treatment of IDD.

### Biomechanical changes in local structures

The instability of the mechanical structure of the IVD is an important cause of complications such as annulus rupture, herniation and nerve extrusion (Adams and Roughley, 2006; Vergroesen et al., 2014; Desmoulin et al., 2020). In recent years, biomechanical changes have been progressively considered as one of the pathological processes of IDD; this is closely related to metabolism, cell renewal and the inflammatory response of IVD tissues (Chan et al., 2011; Neidlinger-Wilke et al., 2012; Desmoulin et al., 2020). Because the IVD is avascular, diffusion is the main transport mechanism used to move nutrients and oxygen (Desmoulin et al., 2020). In addition, due to low oxygen content, IVD cells perform anaerobic respiration which consumes more glucose and produces more metabolic toxicity than aerobic respiration (Bartels et al., 1998). Previous studies have confirmed that excessive loading not only obstructs the diffusion of nutrients and oxygen but also leads to lactic acid accumulation in the NP and AF regions, accompanied by local PH reduction (Huang and Gu, 2008; Wang et al., 2013). Two *in vitro* experiments using human tissues revealed that the diffusion of glucose by AF and CEP diminished with increasing loading (Jackson et al., 2012; Wu et al., 2016). This may be related to the narrowing of the microscopic pore size due to compression. Unfortunately, these results have not yet been verified with *in vivo* data due to a lack of *in vivo* measurement techniques (Desmoulin et al., 2020; Shalash et al., 2021; McDonnell and Buckley, 2022).

Biomechanics also affect cells and their extracellular matrix (ECM). Biomechanical changes affect cellular homeostasis by regulating the expression of several substances, such as matrix metalloproteinases (MMP), cytokines and growth factors (Phillips et al., 2013a; Vo et al., 2013; Neidlinger-Wilke et al., 2014). These substances are bound up with the degradation of the ECM, activation of the inflammatory response and cell loss. Previous studies have shown that tensile strain can cause IVD cells to reduce the expression of proteoglycan and collagen II; these are important for maintaining normal disc performance (Court et al., 2001; Hutton et al., 2002). Svenja et al. (Illien-Jünger et al., 2010) reported that high-frequency loading resulted in cell death and an increase in MMP-13 expression. An *in vivo* study in a mouse model indicated that excessive load exposure resulted in a reduction in tissue inhibitor of matrix metalloproteinase-3 (TIMP-3) and an increase in a disintegrin and a metalloproteinase with thrombospondin motifs-4 (ADAMTS-4); this could relate to an early stage of IDD (Wuertz et al., 2009). It is important to note that a moderate dynamic compressive load is beneficial for IVDs (Walsh and Lotz, 2004; Wang et al., 2007; Paul et al., 2012). In addition, a distinct compressive force on the IVD can cause inflammatory responses; this has been verified in both *in vivo* and *in vitro* models (Wang et al., 2007; Paul et al., 2013; Gawri et al., 2014). Structural deficits in IVDs, such as herniation, can cause immune cell activation and infiltration; these cells include macrophages, neutrophils and T cells (Kokubo et al., 2008; Risbud and Shapiro, 2014). Meanwhile, the loss of intradiscal pressure significantly elevates the release of inflammatory cytokines such as interleukin (IL)-1 and-6 (Dudli et al., 2014). Interestingly, the migration of immune cells into the disc is accompanied by the appearance of nerve fibers arising from the dorsal root ganglion (DRG) (Martínez-Lavín, 2021). These changes are thought to contribute to the sensation of pain. Of note, almost all astronauts experience LBP when the gravity environment changes dramatically, again demonstrating the importance of biomechanics in IVD (Bailey et al., 2018; Lazzari et al., 2021).

### Degradation of the extracellular matrix

Degradation, rebuilding and composition changes are crucial characteristics of IDD pathology. In fact, it is quite common to induce IDD animal models by damaging the ECM, thus demonstrating the importance of the ECM in maintaining IVD stability. The normal ECM consists of two main substances. One is proteoglycans; aggrecan is the most abundant proteoglycan in the NP (Roughley et al., 2006). The polyanionic proteoglycans provide the normal physiological osmotic pressure within the NP, which is crucial in maintaining tissue hydration and buffering compressive forces during daily activities. The water content of the IVD increases from the outer layer of AF to the NP located in the center, as does the concentration of proteoglycans (Feng et al., 2006). In IDD, aggrecan is cleaved from the hyaluronic acid backbone; this reduces its ability to bind water (Yurube et al., 2012; latridis et al., 2011). Another main substance in ECM is collagen. Type I and II collagen are the most abundant collagens in the disc (approximately 80%). The collagen framework of the disc is crucial for preserving normal architecture and function (Roughley et al., 2006). AF cells possess mostly type I while NP cells possess mostly type II collagen. From an anatomical structure viewpoint, from the inside to the outside of the IVD, the content of type II collagen gradually decreases and the content of type I collagen increases (Scott et al., 1994). Previous studies have observed the gradual transformation of notochordal cells into chondrocyte-like cells with increasing age; this reduces the expression of type II collagen and proteoglycans (Scott et al., 1994; Antoniou et al., 1996; Boos et al., 2002; Zhao et al., 2007). Type II collagen has better mechanical compliance than type I; thus, a reduction in type II collagen results in an uneven distribution of stress across the IVD. In addition, the loss of water-binding potential is reflected in the reduction of IVD height (Iatridis et al., 2013; Vergroesen et al., 2014). Collectively, these changes further deteriorate the mechanical environment of the IVD.

Like other substances in the body, normal ECM is in a delicate balance between anabolism and catabolism. Disruption of this balance will undoubtedly lead to the impairment of function. NP cells perform a central role in regulating the anabolism and catabolism of the ECM by regulating the MMP and ADAMTS families of enzymes (Weiler et al., 2002; Vo et al., 2013). Both the excessive activation of MMP and ADAMTS are mainly reflected in the catabolism of ECM. A previous study included 41 specimens of patients who had undergone operations for lumbar disc herniation and analyzed the relationship between the degree of degeneration and the expression of MMP-1 (Xu et al., 2014a). As the degree of degeneration increased, the expression of MMP-1 increased notably. Tang et al. (Tang et al., 2014) used microarray analysis to show that MMP-2 was overexpressed in IDD tissue. The increased expression of MMP-3 was also reported in different animal models (Sobajima et al., 2005; Wei et al., 2014). Furthermore, the upregulation of MMP-7, -8, -10, -12, -13 and-14 were also reported in previous studies (Bachmeier et al., 2009; Richardson et al., 2009; Phillips et al., 2013b; Gruber et al., 2014b; Xu et al., 2014b; Iwata et al., 2015). Similar to MMP, the upregulation of several types of ADAMTS has been reported in degenerated discs (Pockert et al., 2009; Zhang et al., 2012). Of note, Patel et al. (Patel et al., 2007) reported that the amount of ADAMTS-5 in the NP and AF shows no significant difference in different degeneration grades. However, Chen et al. (Chen et al., 2014) reported the upregulation of ADAMTS-5 in the CEPs of IDD patients. Interestingly, ADAMTS-3 and ADAMTS-10 were found to be significantly downregulated in degenerated discs (Gruber et al., 2011). Thus, different tissues may tend to express ADAMTS differently. In addition, the upregulation of inflammatory cytokines, such as IL-1 and tumor necrosis factor (TNF)-α, is closely related to the regulation of MMP and ADAMTS. The upregulation of a variety of catabolic mediators by cytokines, including ADAMTS −4/5, MMP −1, −2, −3, −13 and −14 has been reported in many previous studies (Le Maitre et al., 2005; Séguin et al., 2005; Wang et al., 2011; Shi et al., 2019). The emergence of so many biomarkers makes it possible to better explain the pathological process of IDD and block its pathological progress at an early stage.

### Chronic state of low-grade inflammation

The inflammatory response of IVD tissue and the corresponding release of proinflammatory cytokines are considered important hallmarks of IDD. A prominent feature is the IL-1β and TNF-α secreted by the disc cells (Kadow et al., 2015). These cytokines trigger a range of pathogenic responses, such as amplifying the inflammatory cascade, apoptosis, cellular senescence, pyroptosis and degradation of ECM. Kepler et al. (Kepler et al., 2013b) showed that exposing human NP and AF cells to IL-1β or TNF-α can upregulate the expression of substance P. The contents of other inflammatory factors such as IL-6 and IL-8 were further significantly increased by substance P. In addition, the expression of some other inflammatory molecules was also upregulated by exposure to IL-1β, such as nitric oxide (NO), cyclooxygenase (COX)-2 and prostaglandin E (PGE)-2 (Wang et al., 2019b; Jin et al., 2019). Similarly, the upregulation of IL-17, NO, and PGE-2 in NP and AF cells exposed to TNF-α have also been reported in previous studies (Gabr et al., 2011; Gruber et al., 2013). IL-1β and TNF-α can also regulate chemokine expression in IVD. In a previous study using IVD samples from LBP patients, the expression of C-C motif ligand (CCL)-5 and IL-1β was significantly enhanced in painful IVDs; furthermore, the correlated expression of CCL-5 with IL-1β expression was also indicated (Kepler et al., 2013a). In subsequent *in vitro* experiments, disc cells exposed to both IL-1β and TNF-α upregulated CCL-5 expression, and interestingly, TNF-α promoted higher levels of CCL-5 (Gruber et al., 2014a). Phillips et al. (Phillips et al., 2015) reported that the expression of CCL −2, −3, −4, −5, −6, −7 and C-X-C motif chemokine ligand (CXCL) −1, −8, −9 and −10 was increased by the stimulation of IL-1β. A bioinformatics study reported that the gene expression levels of CCL −3, −20, CXCL −2 and −5 were increased in disc cells under TNF-α stimulation. In addition, IL-1β and TNF-α are closely related to mechanical loading (Elfervig et al., 2001; Wang et al., 2007).

IL-1β and TNF-α also play crucial roles in the regulation of the cell cycle. Yang et al. (Yang et al., 2019) reported that IL-1β stimulated the expression of senescence associated-β-galactosidase (SA-β-Gal), which is considered as a dependable marker of cell senescence. Chen et al. (Chen et al., 2020b) subsequently indicated that IL-1β promoted the progression of IDD, with significantly increased expression of type I collagen, p16, p53 and SA-β-Gal, as well as the reduced expression of type II collagen and aggrecan. Similarly, a previous study from Xie et al. (Xie et al., 2018) indicated that TNF-α could also promote NP cell senescence, reflected by the elevation of SA-β-Gal, p16 and p53 expression. Although apoptosis is important for maintaining cell renewal, once out of balance, excessive apoptosis can lead to the depletion of IVD cells and result in IDD. Jiang et al. (Jiang et al., 2019) demonstrated that IL-1β significantly increased caspase-3 activity, NP cell apoptosis ratio and the expression of Bax, caspase-3, cleaved caspase-3 and cleaved PARP. Similarly, Yu et al. (Yu et al., 2018) reported that TNF-α stimulation markedly improved cell apoptosis ratio, caspase-3 activity, expression of Bcl-2, Bax, and caspase-3, reactive oxygen species (ROS) content, and the activity of the NF-κB pathway.

Pyroptosis is a newfound type of cell death mediated by inflammation. The pyroptosis of cells is often accompanied by an increased number of the NLRP3 inflammasome (He et al., 2015; He et al., 2016). Zhao et al. (Zhao et al., 2021b) reported that an increase in pyroptosis was accompanied by an increase in IL-1β and NLRP3 inflammasome activation. In addition, the disc becomes vascularized and innervated; this has been recognized as an important pathological change of IDD (Karamouzian et al., 2010; Miyagi et al., 2014). The overexpression of vascular endothelial growth factor (VGEF) and neurotrophic factors, such as nerve growth factor (NGF) and brain-derived neurotrophic factor (BDNF), result in vascularization and innervation of the IVD (Liu et al., 2013; Moon et al., 2014; Zhu et al., 2018). Lee et al. (Lee et al., 2011) indicated that IL-1β and TNF-α stimulated the gene expression of VGEF, NGF and BDNF in NP cells; furthermore, they also indicated a positive inter-relationship between IL-1β and VEGF/NGF/BDNF expression using the immunohistochemical method. A deeper understanding of the inflammatory mechanism in IDD will undoubtedly help us to block pathological processes in a timelier manner.

### Cell loss

A sufficient number of cells is undoubtedly necessary to maintain material renewal and the functional maintenance of IVD. Degenerative discs show higher rates of senescence, apoptosis and pyroptosis, thus leading to fewer functioning cells. The most prominent feature of cellular senescence is the irreversible cessation of cell proliferation, which can be identified by the expression of a senescence-associated secretory phenotype (SASP) (McCarthy et al., 2013; Childs et al., 2015; Su et al., 2019). Although multiple molecular signaling pathways are involved in cellular senescence, these all converge on the p53/p21/retinoblastoma (RB) and p16/RB pathways (Chicas et al., 2010; Muñoz-Espín and Serrano, 2014). Because IVD is avascular, this limits the clearance of metabolic waste and immune mediation to cause abnormal accumulation (de Magalhães and Passos, 2018). Previous studies indicated that oxidative stress (OS) and epigenomic perturbations accelerated the cellular senescence of IDD (Campisi et al., 2019). The use of antioxidant drugs to reduce cellular senescence in IDD has been reported in previous studies (Larrañaga et al., 2018; Liu et al., 2018). In addition, genome-wide analysis of DNA methylation profiles revealed significant differences in DNA methylation profiles of NP cells at different stages of IDD. Ikuno et al. (Ikuno et al., 2019) reported 220 differently methylated loci. Unfortunately, differential methylation was found mainly in genes located upstream, which hindered their further clinical application (Ikuno et al., 2019). As key regulators of gene expression, miRNAs have also attracted attention in recent years. Notably, some of these, such as miRNA-338-3p and miRNA-24-3p, are reported to be upregulated in degenerative NP cells (Chen et al., 2020a; Jiang et al., 2021).

Apoptosis and pyroptosis are common modes of programmed cell death and play critical roles in immune regulation. An imperative characteristic of apoptosis is the release of cytochrome-c from mitochondria (Fraser and Evan, 1996). At present, it is commonly accepted that apoptosis mainly includes intrinsic and extrinsic pathways. The dysregulation of intracellular homeostasis by toxic agents or DNA damage triggers the intrinsic pathway; this is characterized by mitochondrial outer membrane permeabilization (MOMP). Under the joint action of MOMP and cytochrome-c, the activity of caspase-3 is upregulated; this is broadly considered the point of no return in apoptotic death (Liu et al., 1996). The activation of cell surface receptors, such as the TNF-related apoptosis-including ligand receptors R1 and R2 triggers the extrinsic pathway (Schneider et al., 1997). Upon activation by their ligands, the intrinsic pathway is finally activated through a complex signaling network (Bertheloot et al., 2021). In the simplified pathway of pyroptosis, caspase-1 is activated by inflammasomes; then, gasdermin-D (GSDMD) is cleaved to GSDMD C-terminal and GSDMD N-terminal. The GSDMD N-terminal can puncture the cell membrane, thus resulting in pyroptosis (Boucher et al., 2018; Wang et al., 2020b). Recent studies demonstrated that (*P. acnes*) is a microbial pathogenic factor of IDD (Berjano et al., 2019). He et al. (He et al., 2020) reported that the expression of NLRP3, caspase -1, caspase -5, and IL-1β were upregulated in NP cells co-cultured with *P. acnes*, thus suggesting the activation of pyroptosis. Tang et al. (Tang et al., 2021a) demonstrated that *P. acnes* activated the pyroptosis of NP cells via the ROS-NLRP3 pathway. The complex relationship between cell loss and other pathological factors makes our understanding of IDD even more challenging, but also provides more directions for subsequent interventions.

## The application of different transmission systems

Our increasing understanding of IDD pathology has led to more therapeutic approaches. Current treatment covers almost all scales of disc tissue from macro to micro levels, such as gene therapy, cell replenishment and surgical treatment (Debono et al., 2018; Li et al., 2021; Roh et al., 2021). Limited by many factors such as ethics, biocompatibility and persistence, IDD therapy, which regulates the metabolism and repairment of cells and tissues, appears to be more popular. Many previous studies have focused on IDD-related drugs, regulatory molecules and gene targets (Chao-Yang et al., 2021; Kamali et al., 2021). Because most of these biologics are fragile and easily decomposed, it is very important to select suitable delivery systems. Nevertheless, comparatively few studies have focused on delivery systems. Here, we exemplify the design principles and application scenarios of delivery systems on different scales.

### Virus vectors

The continuous discovery of IDD-related gene targets makes it possible to intervene in the pathological progress of IDD at the gene level (Cazzanelli and Wuertz-Kozak, 2020). In addition, due to its avascular structure, which makes drug delivery through the blood more difficult, the targeted therapy of IVD cells is a more promising treatment method. As a type of targeted therapy, gene therapy can delay or even reverse IDD by regulating the expression of IDD-related substances in cells (Roh et al., 2021). The method of delivering the proper gene to the target cells is the first issue to be considered. Because of the natural advantages of viruses in delivering genetic information to host cells, viral vectors have attracted extensive attention.

**FIGURE 2:**
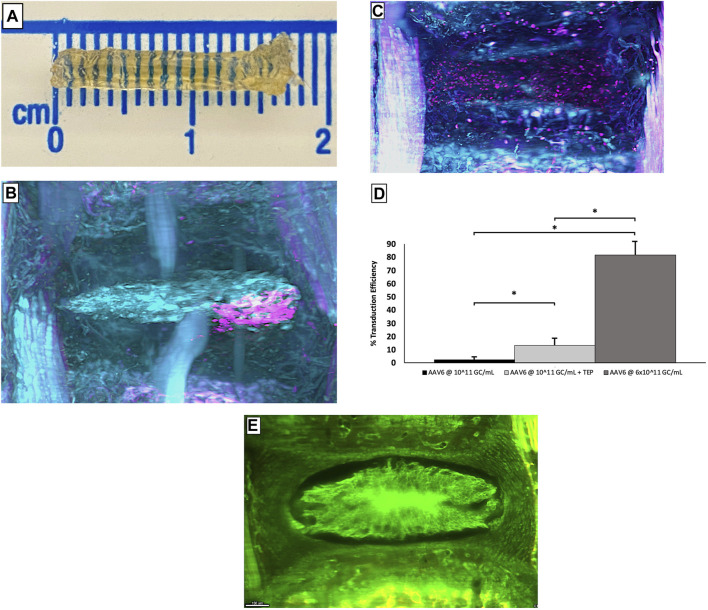
Complete mice caudal spines obtained using the PEGASOS method **(A)**. The images of the caudal tissues transferred by AAV6 alone **(B)** and AAV6+cell-permeable peptide **(C)** are viewed using 3D lightsheet microscopy, pink represents cells that transduced successfully, and cyan represents cells that transduced failed. Statistical analysis of the number of NP cells successfully transduced under each condition **(D)**. The representative image of IVD tissue without punctured **(E)**. Error bars show SEM. ‘∗’ means *p* < 0.05, indicating a statistical difference. Reprinted permission by CC BY license (Kim et al., 2022). https://creativecommons.org/licenses/by-nc-nd/4.0/.

Retroviruses were the first to be considered. Wehling et al. (Wehling et al., 1997) successfully transfected a target gene by retroviruses into chondrocytes from bovine CEP *in vitro*. Unfortunately, the effect of retroviruses on cells with a low-dividing rate is restricted, thus limiting the use of retroviruses in IDD (Bartels et al., 1998; Desmoulin et al., 2020). Subsequently, researchers turned to adenoviruses because of their higher transduction efficiency, notably in quiescent or slow-dividing cells (Moon et al., 2000). Many previous studies have demonstrated that adenovirus delivery systems play a significant role in regulating inflammation and improving ECM metabolism (Liang et al., 2010; Luo et al., 2016; Zheng et al., 2018). However, the potential infectivity and immunogenicity of adenoviral vectors cannot be ignored. In 1999, the first case of lethal systemic inflammation after the intra-arterial injection of an adenovirus vector was reported. This has forced researchers to stop the rapid development of adenoviral vectors and reassess their safety. In a safety assessment of gene transfer for the treatment of IVD, Wallach et al. (Wallach et al., 2006) indicated that improperly dosed or direct gene delivery to disc tissue may result in adverse complications. Driesse et al. (Driesse et al., 2000) reported that recombinant adenovirus may cause infection of the central nervous system. In fact, there has been a significant decline in the number of articles investigating adenoviral vectors over recent years; these have been replaced by adeno-associated viruses (AAVs). Compared with adenoviruses, AAVs have lower immunogenicity and safety has been confirmed (Levicoff et al., 2008; Mern et al., 2017). In 2015, Mern et al. (Mern and Thomé, 2015) identified NP cell-specific AAV serotypes by transfecting human NP cells with diverse AAV serotypes. This study makes AAVs more promising in the treatment of IDD. Kim et al. (Kim et al., 2022) reported that AAV6 could provide superb transduction efficiency with limited cytotoxicity *in vivo* (Figure 2). Although some AAV-based therapeutics gained regulatory approval in Europe or the United States, their use in IDD has been limited by their high price and difficulties in guaranteeing homogeneity between different batches (Wang et al., 2019a). Lentiviruses have become popular vectors in recent years, mainly due to their ability to transduce non-dividing cells. Wu et al. (Wu et al., 2014) reported that lentiviral vector-mediated gene expression lasted nearly twice as long as AAV in human NP cells. The regulation of IDD-related factors such as inflammation, ECM and apoptosis by lentiviral vectors has been reported (Farhang et al., 2019; Lin et al., 2021; Lu et al., 2021; Lu and Lin, 2021).

Although virus vectors have great potential, the high development cost, the need for rigorous toxicology tests, safety verification, the lack of standardization, and inherent batch-to-batch differences are all difficulties that need to be overcome.

### Physical methods

**FIGURE 3:**
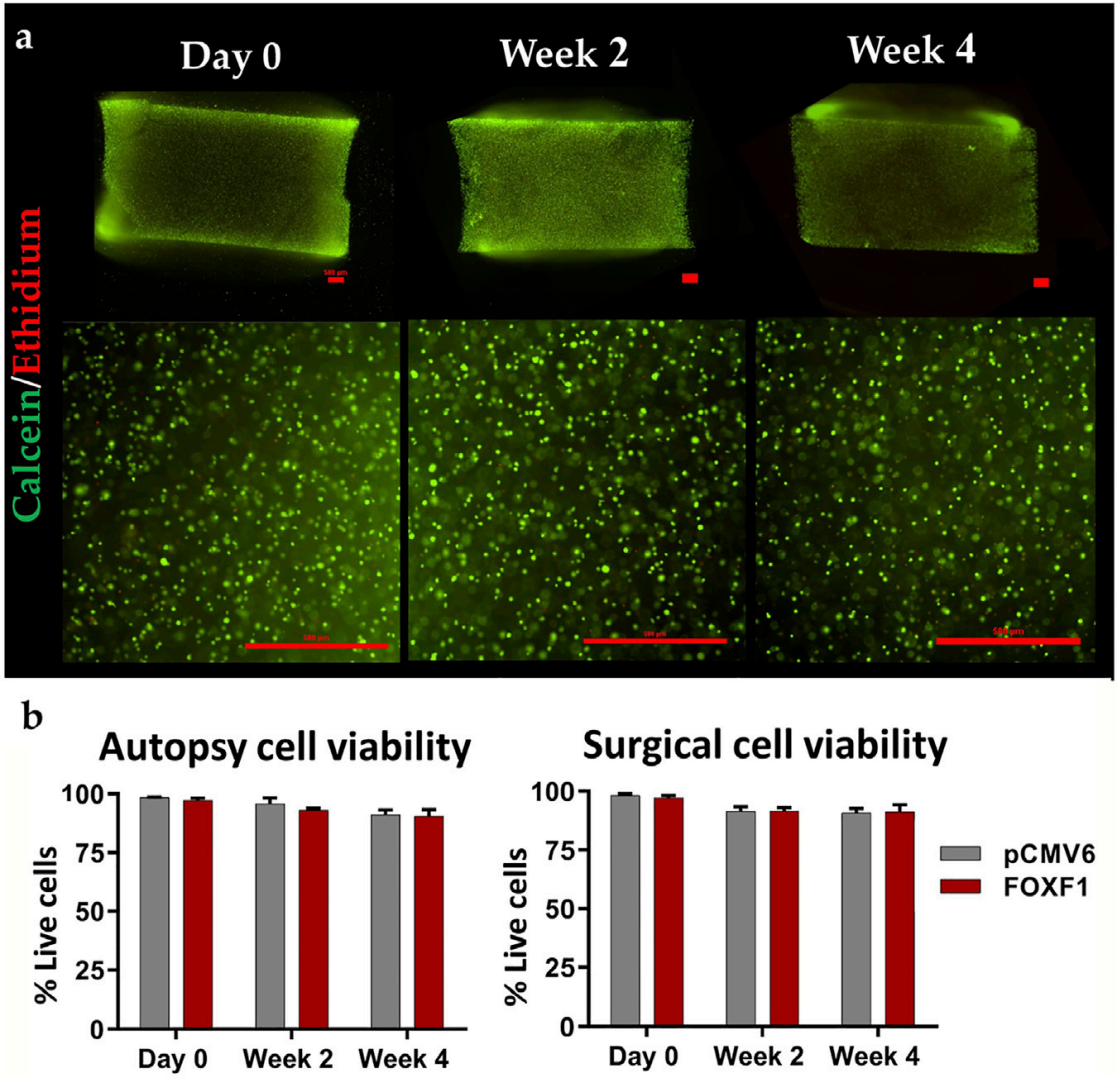
Representative 4 × (top) and 10 × (bottom) stained images (scale bar: 500 μm) of gel sagittal frozen sections containing human NP cells at day 0, week 2 and 4 weeks in culture **(A)**; green represents cells that survived after electroporation transduction, and red represents cells that died after electroporation. The live rate of cells transfected with FOXF1 and pCMV6 by electroporation in the autopsy group and surgery group were quantified **(B)**. Reprinted permission by CC BY license (Tang et al., 2021c). https://creativecommons.org/licenses/by-sa/4.0/.

Rather than using a virus as a transit point, physically delivering the target material into a cell seems to be a more straightforward solution. The main options at present are ultrasound exposure and electroporation. In 2006, Nishida et al. (Nishida et al., 2006) established a novel gene therapy technique for IVD cells using ultrasound. The target gene was wrapped in microbubbles, and these microbubbles were injected into IVDs. Immediately after injection, ultrasound was irradiated on the surface of the injected discs, thus resulting in the breaking of the microbubbles and transient holes in the cell surface (Nishida et al., 2006). Electroporation is a method that uses short electrical pulses to disturb cell membranes and momentarily creates holes in the membranes that allow genetic material to pass through. Once the optimal conditions for electroporation are determined, it is possible to transfer many cells in a short period. May et al. (May et al., 2017) indicated that two pulses of 1400 V for 20 ms resulted in favorable and reproducible results for both human and bovine IVD cells. Tang et al. (Tang et al., 2021c) used the electroporation method to deliver forkhead-box F1 mRNA to degenerated NP cells in human disc tissue and significantly reduced the secretion of IL-1β and MMP-13 (Figure 3). In addition, laser-mediated transfection and magnetofection are also used in other diseases (Antkowiak et al., 2013; Sizikov et al., 2021). Despite physical methods that can deliver genetic materials directly into target cells, there are non-negligible drawbacks, such as lower transfection rates, potential cell damage, and difficulty in determining the appropriate stimulation intensity.

### Hydrogel-based delivery systems

Hydrogel is a three-dimensional mesh polymer with a hydrophilic structure that is able to bound large amounts of water. A microenvironment with ideal water content is beneficial to maintain the metabolism of IVD tissue and promote regeneration. Besides water, hydrogels can also provide mechanical support for cell proliferation. Hydrogels are highly manipulable because their mechanical strength, degradability, gelation time/temperature and other characteristics can be modified. Due to these advantages, hydrogels have been widely used to intervene with the pathological development of IDD.

There are various methods to arrange hydrogels into different subclasses. At the most fundamental level, natural hydrogels and synthetic hydrogels are classified according to their source composition. Natural hydrogels such as cellulose, alginate and hyaluronic acid always have favorable biocompatibility and low toxicity. However, most natural hydrogels can barely meet the mechanical stress requirements needed in human IVD tissue, thus severely limiting their application (Growney Kalaf et al., 2017; Alinejad et al., 2019). Compared with natural hydrogels, synthetic hydrogels generally have less variability and greater flexibility in mechanical properties, although their potential toxicity to cells cannot be ignored (Klein et al., 2009). As the name suggests, composite hydrogels are composed of at least two components and seem to overcome defects caused by a traditional single component. According to the cross-linking form, composite hydrogels can be classified into chemical and physical hydrogels. Chemical hydrogels are linked by covalent bonds, making the cross-linking irreversible, while physical hydrogels are connected by molecular entanglement and secondary forces and the transformations are reversible (Hoffman, 2002). Nevertheless, in the clinical transformation of hydrogels, it is difficult to implant gelatiniform hydrogels *in vivo* due to the deep target location and complex anatomy.

It is worth noting that traditional *in situ*-forming bulk hydrogels have relatively low efficiency in exchanging substances with tissues. In fact, exchanges with the target microenvironment are limited to the hydrogel/tissue interface. In recent years, stimulus-responsive composite hydrogels have been developed rapidly. Because stimulus-responsive composite hydrogels can solidify rapidly only after receiving the corresponding stimulus, they can be injected into the damaged area in a sol-gel state, thus making the hydrogels fit the tissue well. There are various types of stimuli, such as physical, chemical and biological (Koetting et al., 2015; Choi et al., 2019). Considering the changes in the pH microenvironment caused by inflammation and dysregulation of cellular metabolism, pH-sensitive composite hydrogels seem to have ideal prospects. The ionizable pendant groups in the backbone of the hydrogel are responsible for its pH-sensitive behavior (Gupta et al., 2002). According to pendant group ionization, pH-sensitive composite hydrogels can be classified into anionic hydrogels and cationic hydrogels. The generation of an electrostatic repulsive force leads to the swelling and deswelling of the hydrogel; this is accompanied by the absorption and expulsion of water (Gupta et al., 2002). Nguyen et al. (Nguyen et al., 2019) reported the synthesis of a type of poly (ethylene glycol) (PEG)-based hydrogel with adjustable mechanical strength via ring-opening reactions; cytotoxicity tests showed that human NP cells remained a high survival rate after 8 days. In another previous study, Zhang et al. (Zhang et al., 2022b) adjusted the ratio between components to reach a balance of histocompatibility and mechanical stability of poly (acrylic acid) (PAA) hydrogels. Collectively, the most common monomers that can be used to introduce pH-sensitive properties include methacrylic acid (MMA), acrylic acid (AA) and dimethylaminoethyl methacrylate (DMAEMA) (Koetting et al., 2015). In addition, some natural hydrogels can also exhibit pH-sensitive properties, such as albumin and alginate (Zheng and Du, 2021). Considering the constant temperature inside the body compared to outside, temperature-sensitive composite hydrogels have also attracted the attention of researchers. Temperature-sensitive composite hydrogels can be divided into two fundamental subtypes: positively and negatively responsive hydrogels. Common monomers with temperature sensitivity include cellulose derivatives, chitosan, PEG and poly (N-isopropyl acrylamide) (pNiPAAm) (Klouda and Mikos, 2008). Schmitt et al. (Schmitt et al., 2021) reported the long-term evaluation of an injectable chitosan carboxymethyl cellulose hydrogel in an ovine model. Compared with the untreated group, the intervertebral disc was stabilized, and the progression of degeneration was significantly slower in the treated group after 12 months. Furthermore, due to its low cytotoxicity and excellent biocompatibility, PEG has been widely used in medical applications such as promoting the healing of surgical incisions or drug delivery (Tan et al., 2019; Chen et al., 2022).

**FIGURE 4:**
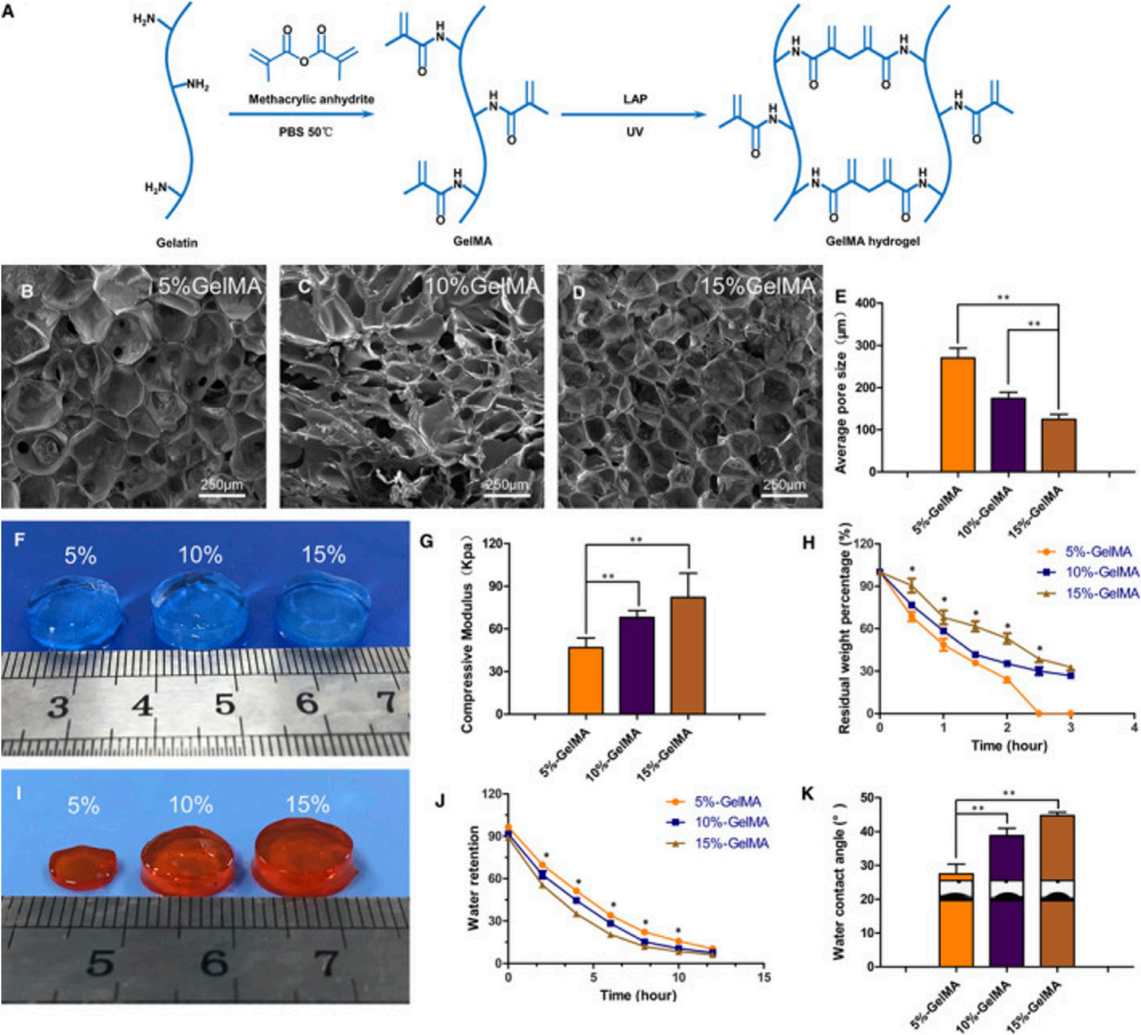
Schematic diagram of synthetic molecules of GelMA hydrogels **(A)**. Scanning electron microscopy was used to observe the microstructure of GelMA hydrogels with different concentrations **(B–D)**. Comparison of the pore size of hydrogels with different concentrations **(E)**. Comparison of the appearance of three different concentrations of hydrogels **(F)**. Comparison of mechanical properties of three different concentrations of hydrogels **(G)**. Degradation of hydrogels with different concentrations over time *in vitro* **(H)**. The appearance of hydrogel after 2 h *in vitro* degradation **(I)**. Comparison of water binding capacity of hydrogels with different concentrations **(J)**. Comparison of water content angle of hydrogels with different concentrations **(K)**. (*p < 0.05, **p < 0.01). Reproduced with permission from (Xu et al., 2021).

A significant advantage of photosensitive composite hydrogels is easy to access to light stimulation and the precise control of stimulation. Thus far, three types of photosensitive monomers have attracted a lot of attention, including gelatin methacrylate (GelMA), photopolymerized polyethylene glycol diacrylate (PEGDA) and hyaluronic acid methacrylate (HAMA) (Burdick and Prestwich, 2011; Clark et al., 2017; Kurian et al., 2022). Xu et al. (Xu et al., 2021) combined collagen hydrolysate and GelMA to make a photosensitive composite hydrogel that could encapsulate NP cells *in vitro* (Figure 4). Unfortunately, the debilitating effect of IVD tissue on light stimulation prevents the application of photosensitive composite hydrogels *in vivo*. Considering that there are some upregulated enzymes in IDD, such as MMP, some enzyme-sensitive composite hydrogels focusing on IDD-related enzymes have been developed in recent years, although more *in vivo* experiments are needed (Skaalure et al., 2015; Amer and Bryant, 2016; Schneider et al., 2020). Due to the special mechanical and metabolic environment of the degenerated disc, pressure-sensitive composite hydrogels and ion-sensitive composite hydrogels are expected to play a role in IDD treatment; however, few relevant studies have been reported.

There has been promising progress in stimulus-responsive composite hydrogels, although the advances in biological material and tissue engineering technologies have led researchers to focus on more microscopic structures. A better understanding of microstructure would allow us to better manipulate the properties of materials.

### Microsphere-based delivery systems

Just as stimulus-responsive composite hydrogels solve the problem of tissue fitting when compared to *in situ* gels, injectable microsphere-based delivery systems can provide superior substance exchange efficiency. Microspheres are a class of three-dimensional spherical structures with a mean particle diameter of 1–1,000 μm (Gupta et al., 2017). According to the distribution of therapeutic substances in microspheres, microspheres can be divided into microcapsules and micromatrices. Microcapsules take the therapeutic substance as the core, while micromatrices are formed when the therapeutic substance is dispersed or embedded in the entire material matrix (Kulchar et al., 2021). Due to its good delivery capacity and biocompatibility, the microsphere system has significant application potential as a cell, drug and biological factor delivery system in IVD therapy. Common natural ingredients include collagen, chitosan, gelatin and alginate. Indeed, gelatin- and collagen-based microsphere systems are already commercially available (Chen et al., 2013).

**FIGURE 5:**
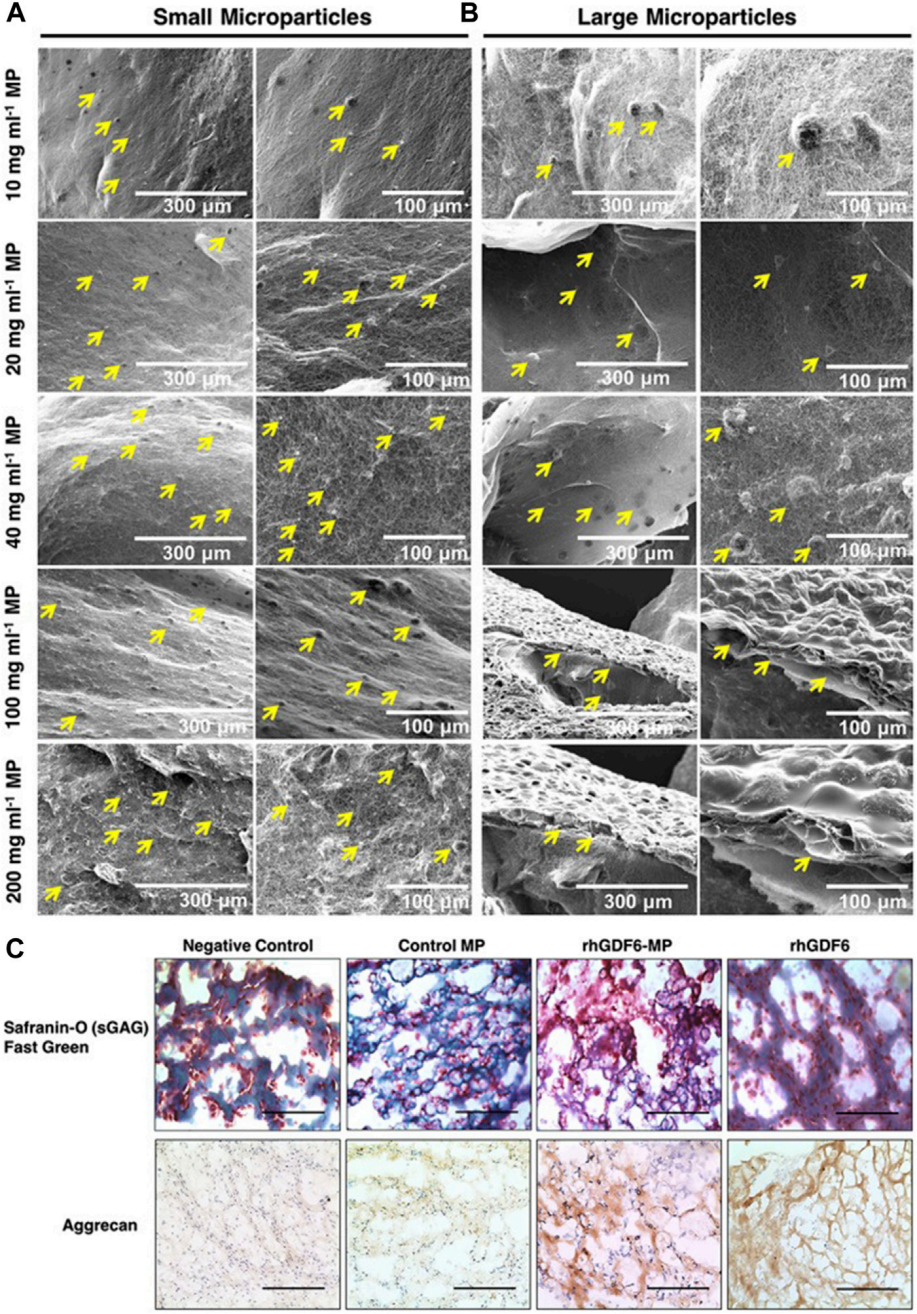
The binding ability of different concentrations of small **(A)** and large **(B)** microspheres to collagen gel under scanning electron microscopy. After 14 days of culture, the microspheres containing rhGDF-6 showed more ideal target substances expression in both histological and immunohistochemical staining **(C)**. Scale bars = 300 μm. Reproduced with permission from (Hodgkinson et al., 2019).

Poly (lactic-co-glycolic acid) (PLGA) copolymer is one of the most widely used biomaterials at present due to its excellent biodegradation and biocompatibility (Zhao et al., 2021a; Zhu et al., 2022a). PLGA is synthesized by lactic acid (LA) and glycolic (GA) through random polymerization (Su et al., 2021). Hodgkinson et al. (Hodgkinson et al., 2019) reported that recombinant human growth difference factor (rhGDF-6) was loaded in PLGA-PEG-PLGA microspheres and induced the differentiation of adipose stem cells (ASCs) to NP cells and aggrecan production (Figure 5). Huang et al. (Huang et al., 2021) used PEG-PLGA-PGE as a drug carrier and showed that this microsphere system had ideal biocompatibility and low cytotoxicity. In a recent study, Lim et al. (Lim et al., 2022) encapsulated ABT263, a senolytic drug, in PLGA microspheres which were intradiscally administered into injury-induced IDD rat models. Analysis showed the selective clearance of senescent cells from degenerative IVD tissue, the downregulated expression of proinflammatory cytokines and the restoration of the IVD structure. However, considering the more complex anatomical structure and mechanical environment of the human spine, the application of PLGA microspheres in human IVD still needs to be demonstrated in further experiments.

Poly (ε-caprolactone) (PCL) is a polymer with very low glasses transition temperature and melting point. Due to its good biocompatibility, PCL has been widely used in medicine; for example, as surgical sutures (Wei et al., 2009). Jang et al. (Jang et al., 2020) embedded hyaluronic acid (HA) in PCL/HA hybrid microspheres, and the results indicated that the PCL/HA hybrid microspheres provided a proper environment for the proliferation and differentiation of human periosteum-derived cells *in vitro*. Zhou et al. (Zhou et al., 2020) presented a simple method that combined poly (vinyl alcohol) (PVA) hydrogels with PCL and reflected a new type of microsphere with a PVA core and PCL shell. Unfortunately, due to its slow decomposition *in vivo*, PCL is mainly used as a scaffold material to repair AF or osteonecrosis, and its use as a microsphere-based delivery system in IDD has been rarely reported (Shamsah et al., 2019; Zhang et al., 2022a).

Since its release properties can be modified by adjusting physical and chemical parameters, researchers can better regulate the release of therapeutic substances by microsphere hydrogels. Chang et al. (Chang et al., 2022b) reported a circRNA silencing-hydrogel microsphere by grafting silencing genes loaded on HAMA microspheres. The results indicated that HAMA microspheres had ideal degradability and that silencing genes can be significantly released for 27 days. Platelet-rich plasma (PRP) is the product of whole blood centrifugation and can relieve IDD symptoms in humans (Tuakli-Wosornu et al., 2016). In another previous study, Miran et al. (Choi et al., 2020) reported that loading powdered PRP into PEG microspheres could significantly prolong the degradation and protein release time of microspheres. In short, the development of microsphere hydrogels brings us one step closer to the single injection of drugs that could provide long-term intervention for IDD processes.

As a common delivery system, the microsphere-based delivery system has a broad clinical application prospect for IDD. However, rigorous safety audits must be carried out and the performance degradation caused by the complex physical and chemical environment in the human body must be considered. In addition, the parameters that are more suitable for IVD tissue also require extensive preliminary experiments.

### Nano-scale delivery systems

Besides microspheres, many nanomaterials are also used for drug delivery because they are more easily taken up by cells. Therapeutic substances can be immobilized on or encapsulated by such nanomaterials. Nanomaterials that can be degraded *in situ* are generally considered to have excellent biocompatibility and low immunogenicity (Mitchell et al., 2021). Common nanomaterials used for nanocarriers include lipids, polymers and inorganic compounds.

**FIGURE 6:**
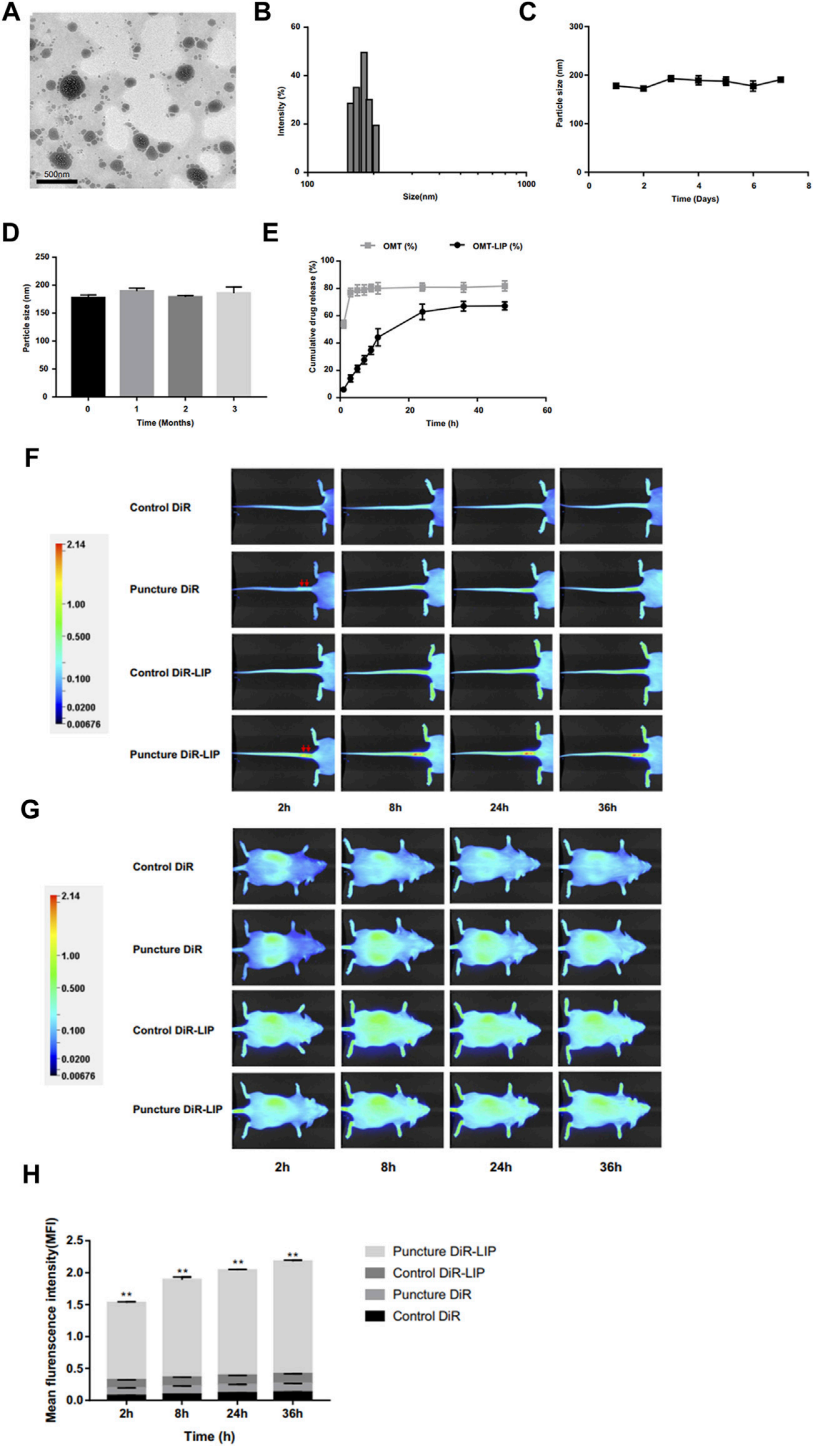
Image of oxymatrine liposome staining under the transmission electron microscope **(A)**. The particle size distribution of oxymatrine liposome **(B)**. The particle size changes of oxymatrine liposome during storage for 7°days **(C)** and 3°months **(D)** at 4°C. The curve of oxymatrine liposome releasing oxymatrine *in vitro* **(E)**. The NIFR images of the liposome delivery system biodistribution in tails **(F)** and whole bodies **(G)** at different timepoints (2, 8, 24, and 36 h). Red arrows indicate puncture sites. Statistical analysis of fluorescence intensity at the degenerative discs **(H)**. (**p < 0.01). Reproduced with permission from (207). https://creativecommons.org/licenses/by-nc/3.0/.

Lipid-based delivery systems have many inherent advantages, such as simple composition, good biocompatibility, low toxicity and self-assembly (Sercombe et al., 2015). Liposomes are the most well know lipid-based delivery systems. In addition, some artificial liposomes also show promising therapeutic prospects. Banala et al. (Banala et al., 2019) were able to reduce the expression of caspase-3 and ADAMTS-5 via liposomes loaded with siDNA. In 2020, Wang et al. (Wang et al., 2020a) used liposomes to deliver oxymatrine and found that the degeneration of IDD was slowed down both *in vitro* and *in vivo* (Figure 6). It is worth noting that liposomes are susceptible to the interference of complex environments *in vivo*, thus limiting their clinical application. Extracellular vesicles (EVs) can break through the limitations of mesenchymal stem cells (MSCs) in the treatment of IDD, of which exosomes have received widespread attention over recent years. Kang et al. (Lu et al., 2017) reported that exosomes act as a critical transport agent in information communication between MSCs and NP cells. Luo et al. (Luo et al., 2021) confirmed that CEP delayed the progression of IDD via exosomes. Similarly, Sun et al. (Sun et al., 2021b) reported that the endothelial cell migration and inflammation response could be controlled by AF cell-derived exosomes. EV-based therapies are also developing rapidly. Liao et al. (Liao et al., 2021) achieved a high uptake of EVs loaded with antioxidant proteins in TNF-α-treated NP cells by gene-editing parental MSCs; this resulted in reduced cell death and lower progression of IDD. In a subsequent study, Qian et al. (Qian et al., 2022) reported that the application of PRP-derived exosomes could alleviate IDD-associated inflammation. At present, the optimal dose and mode of administration of exosomes are not clear, and due to their cell origin, they may receive a more stringent ethical review.

Polymeric nanoparticles can be classified into three types: polymersomes, micelles and dendrimers. Similar to liposomes, polymersomes have membranes made by amphiphilic block copolymers. Polymersomes are generally more stable and less permeable than liposomes, but many factors can affect these properties, such as the diameter of the polymeric vesicle and the inherent properties of the material (Rideau et al., 2018). Polymersomes have been extensively studied in the fields of anti-inflammation and tumors and their application in IDD is worth considering. Polymeric micelles have a hydrophilic core and a hydrophobic coating and have been used in the clinical treatment of ovarian cancer (Lee et al., 2018). Zhang et al. (Chang et al., 2022a) used polymeric micelles loaded with mRNA to improve the synthesis of ECM. Dendrimers are branched polymers, and their size, mass, and shape can be tightly controlled (Mitchell et al., 2021). Dendrimers have been widely used in many clinical applications, such as contrast media and topical gels (Kannan et al., 2014; Palmerston Mendes et al., 2017). However, the further use of polymeric nanoparticles in human bodies requires strict safety reviews.

Inorganic materials such as carbon and silicon can also be used to establish nano-scale delivery systems. Carbon nanotubes are hollow graphitic structures made of a layer of graphene; therefore, they have a high surface area and excellent electrical conductivity (Negri et al., 2020). Carbon nanotubes have an excellent ability to penetrate cell membranes and bind therapeutic substances. Carbon nanotubes have significant potential in the diagnosis and treatment of cancer and neurodegeneration (John et al., 2015; Tang et al., 2021b). In addition, carbon nanotubes modified with hydrogels have better mechanical, physical and biological features (Vashist et al., 2018). All of these findings provide a reference for the application of carbon nanotubes in IDD. However, the clinical application of carbon nanotubes still is still associated with difficulties that need to be overcome, such as poor water solubility, low biodegradation and dispersion. There have been a number of studies focusing on the use of mesoporous silica nanoparticles in cancer-related drug systems (Li et al., 2019). Mesoporous silica nanoparticles exhibit many advantages such as a large surface area, a high pore volume, tunable pore size and excellent stability. Wang et al. (Wang et al., 2020c) reported the synthesis of mesoporous silica nanoparticles with a cell-targeting function. Although mesoporous silica nanoparticles have broad application prospects, it is necessary to adjust parameters such as hollow structure, surface chemistry and pore size based on IVD tissue and corresponding drug properties; this requires a large amount of *in vivo* and *in vitro* experiments.

The application of nanotechnology provides a new solution for the treatment of IDD but still needs to be personalized and adjusted according to the local complex microenvironment in the spine. In addition, the potential cytotoxicity requires further attention.

### Multiscale delivery systems

With the rapid development of new technologies such as gene programming and bio-3D printing, the boundaries between biology, materials science, pharmacy and other related disciplines are gradually becoming blurred (Cui et al., 2020; Zhu et al., 2020). Multi-disciplinary and multi-scale technology exchanges make up for the shortcomings of a traditional single technology, which undoubtedly brings new hope for the treatment of IDD. Multi-scale delivery systems do not only include the multiscale aspect; here, we prefer to extend this concept to the temporal scale in which this type of system could show difference performance in different disease stages.

**FIGURE 7:**
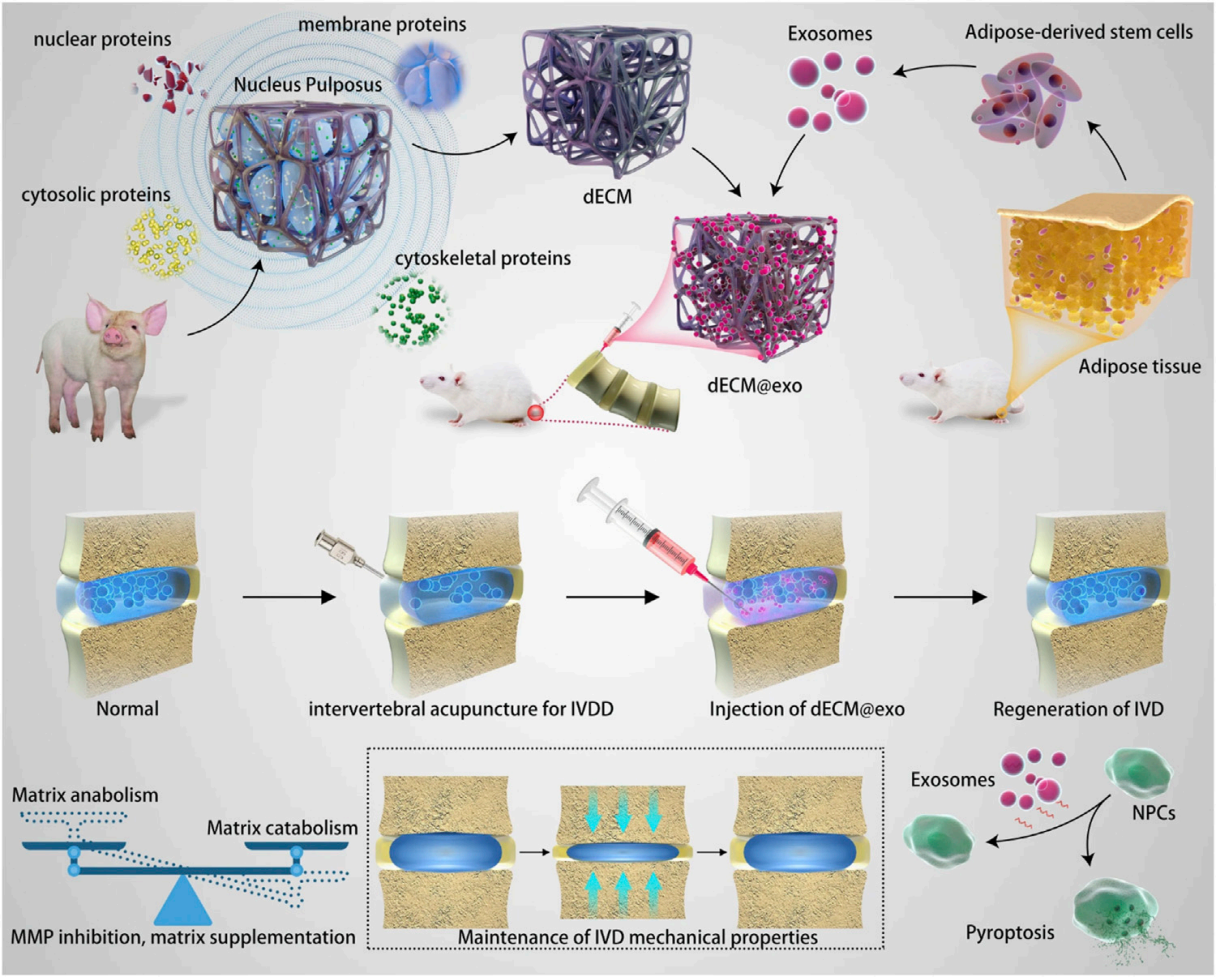
Schematic diagram of the mechanisms of one kind of multiscale drug delivery system combining thermosensitive acellular ECM hydrogels and adipose-derived MSC exosomes for IVD treatment. Reproduced with permission from (Xing et al., 2021).

The pathological process of IDD is a multi-scale process from the gene to the local anatomical structure, which makes it difficult to obtain satisfactory results by intervening with a single factor; therefore, the establishment of a multi-scale delivery system has good prospects. Ligorio et al. (Ligorio et al., 2019) reported a new hybrid injectable 3D-scaffold that used graphene oxide (GO) as a nanofiller; results indicated that the 3D-scaffold had similar mechanical properties as health NP tissue and promotes metabolic activity. In addition to its potential to deliver NP cells, researchers explored the possibility of using the 3D-scaffold as a transporter that modulated the sequestration and delivery of transforming growth factor beta-3 (TGF-β3) (Ligorio et al., 2021). In their latest study, these authors extended the use of the 3D-scaffold as a versatile pH-tunable platform for cell encapsulation (Ligorio et al., 2022). Instead of using one system to interfere with pathological processes at different levels, it seems more convenient to combine two systems that work at different levels as one. Due to the instability of exosomes *in vivo*, their clinical application has stalled. Zhou et al. (Xing et al., 2021) reported the combination of thermosensitive acellular ECM hydrogels and adipose-derived MSC exosomes. This hydrogel system can provide both gelation for ECM leakage and a suitable environment for the growth of NP cells (Figure 7). In addition, sustained released exosomes significantly downregulated the expression of MMP and mitigated the inflammatory response *in vitro.*

Bio-3D printing is an emerging technology platform that uses computational methods to precisely manipulate the distribution of biomaterials, biological factors and cells. Due to its flexible, precise, and personalized features, bio-3D printing can meet people’s needs more accurately than traditional manufacturing methods (Kang et al., 2016). Because printing different materials requires different parameters, it is difficult to integrate biomaterials, biological factors and cells into one system. Sun et al. (Sun et al., 2021a) designed a new scaffold with biomaterials, cells and biological factors. The replica NP component was printed from MSCs and TGF-β3 hydrogel while the replica AF part was printed by MSCs and connective tissue growth factor hydrogel. In addition, the framework of the whole scaffold was printed by PCL to provide necessary mechanical support (Sun et al., 2021a). Zhu et al. (Zhu et al., 2021) conceived another method to solve this problem by using poly lactide (PLA) to print an IVD frame structure; AF and NP are modeled by oriented porous poly (l-lactide)/octa-armed polyhedral oligomeric silsesquioxanes fiber bundles and gellan gum/PEGDA hydrogel loaded with MSCs, respectively. Both scaffolds exhibited good mechanical properties *in vitro*, maintained high cell activity and produced new ECM *in vivo*.

A sequential delivery system that can deliver corresponding drugs at different pathological stages in the temporal dimension is also remarkable. Bayer et al. (Bayer et al., 2017) established a delivery system that achieved the sequential release of platelet-derived growth factor (PDGF) and bone morphogenetic protein-2 (BMP-2) by combing alginate matrices with calcium phosphate scaffolding. In a subsequent study, Leslie et al. (Frapin et al., 2020) developed a delivery system based on pullulan microbeads. This sequential delivery system first released CCL-5 to recruit stem cells to NP, followed by the release of TGF-β1 and growth/differentiation factor (GDF)-5 to upregulate the expression of type II collagen and aggrecan.

New technologies and multidisciplinary communication have accelerated the development of multi-scale delivery systems, but long-term safety assessment and adequate *in vivo* experiments on large mammals still need to be carried out. In addition, the clinical experience and the need for detailed evaluation also need to be considered.

## Conclusion

IDD is an important health problem that has a significant effect on a patient’s quality of life. In today’s aging society, this problem should be given increased levels of attention. Although we are still exploring the detailed pathological process of IDD, we have reviewed several widely accepted mechanisms in the development of IDD; these mechanisms are interconnected and form a vicious loop. Treatment methods for these mechanisms are also developing rapidly, covering almost every time stage and physical scale of IDD development. Although significant levels of attention have been paid to specific targets and effective drugs, an equally important question tends to be overlooked: how do we get these therapeutic substances to where they should be and in the way that people expect them to be? In this review, we summarized the pathological mechanisms associated with IDD and described the delivery systems at various scales. We emphasized the multi-scale delivery systems brought about by multidisciplinary communication as a new direction for delivery systems. However, there are still many difficulties to be overcome in the selection of suitable carriers. Cytotoxicity, degradability, and biocompatibility are unavoidable problems for all implants. In addition, the technical difficulty and cost of materials are also practical problems that need to be considered. The choice is often a trade-off between various performances, and it is difficult to meet all the expectations at the beginning of the design.

When the delivery system is considered for clinical application, it is necessary to discuss the practical aspects; various delivery systems are briefly evaluated in Table 1. The mechanisms responsible for IDD and LBP remain unclear and some IDD patients do not receive special examinations before the onset of symptoms. How we screen early-stage IDD patients and persuade them to accept treatment is the first problem that clinicians need to address. The next problem is the personalization of therapeutic strategies and how to select the appropriate system according to the specific condition of patients; this requires more research. Finally, any material implanted into the body should undergo rigorous safety assessments and pre-clinical validation.

**TABLE 1:** A brief comparison of various delivery systems.

Feature	Delivery palace	Substance carried	Major advantages	Major disadvantages
Virus vector				
retroviruses	intracellular	genetic information	Wide range of sources, simple to operate and easy to build	Weak effect on low-dividing rate cells
adenoviruses	intracellular	genetic information	Higher transduction efficiency in quiescent slow-dividing cells	Potential infectivity and immunogenicity
adeno-associated viruses	intracellular	genetic information	Biocompatible, low immunogenicity	Costly and difficult to ensure homogeneity between batches
lentiviruses	intracellular	genetic information	Ability to transduce nondividing cells	Costly, low manufacturing capacity and complex bioprocessing
Physical method				
ultrasound	intracellular	genetic information/drug molecular	Simple procedure and no immunogenicity	Potential damage to cells
electroporation	intracellular	genetic information/drug molecular	Ability to transfer a large number of cells in a short period	Potential damage to cells and difficulty in determining the appropriate stimulation intensity
Hydrogel-based delivery system				
natural polymers	extracellular	genetic information/drug molecular/cell	Good biocompatibility, biodegradable, some are biological functional	*In vivo* performance is difficult to meet the requirements, easy to decompose
synthetic polymers	extracellular	genetic information/drug molecular/cell	Better mechanical properties and easy to ensure stable performance between batches	Possible cytotoxicity and low biocompatibility
stimulus-responsive composite hydrogel	extracellular	genetic information/drug molecular/cell	Adjust performance parameters based on requirements	Potential cytotoxicity, design difficulties and extensive pre-testing for ideal parameter
Microsphere-based delivery system				
microcapsule	Intracellular/extracelluar	genetic information/drug molecular/cell	Superior substance exchange efficiency, the parameters can be adjusted, targeted drug delivery	Complicated fabrication methods, have some requirements for the loading
micromatrice	Intracellular/extracelluar	genetic information/drug molecular/cell	Better compatibility with different loading, higher encapsulation rate, targeted drug delivery	Ideal parameters require extensive per-testing, complicated to design
Nano-scale delivery system				
Lipid-based delivery system	Intracellular/extracelluar	genetic information/drug molecular	Simple composition, good biocompatibility, low toxicity, and some are self-assembly	Susceptible to the interference of complex environment *in vivo*
Polymeric nanoparticles	Intracellular/extracelluar	genetic information/drug molecular	Good stability, adjustable parameters	Require rigorous safety assessment, complicated to design
Inorganic material	Intracellular/extracelluar	genetic information/drug molecular	Good stability, biocompatibility, and low toxicity	Difficult to decompose, ideal parameters require extensive per-testing
Multiscale delivery system	Intracellular/extracelluar	genetic information/drug molecular/cell	Multiscale intervention, more lasting and effective treatment	High cost, complicated to design

In conclusion, we should design delivery systems with better theoretical performance through multidisciplinary and advanced technology. Furthermore, we should adjust the parameters and components of these systems according to the specific situation of patients. Although a significant amount of research is still needed, we still expect that multi-scale delivery systems will bring new opportunities for IDD treatment.
